# Metals and Metalloids Increase in *Cycas micronesica* Seed Gametophyte Tissue in Shaded Growth Conditions

**DOI:** 10.3390/toxics10100550

**Published:** 2022-09-20

**Authors:** Thomas E. Marler, Christopher A. Shaw

**Affiliations:** 1 Bagong Kaalaman Botanikal Institute, 15 Rizal Street, Barangay Malabañas, Angeles City 2009, Philippines; 2 Department of Ophthalmology and Visual Sciences, University of British Columbia, Vancouver, BC V5Z 1M9, Canada

**Keywords:** aluminum toxicity, ALS-PDC, Guam, metal distribution

## Abstract

Exposure to environmental toxins may be partly responsible for mammal neurodegenerative disorders. Consumption of seeds from Guam’s cycad tree has been linked to the disorder known as amyotrophic lateral sclerosis-parkinsonism dementia complex (ALS-PDC). The unambiguous identification of causal agents of ALS-PDC has been elusive. We have examined the levels of eight metals and metalloids in cycad seeds as a function of the ambient shade in which the plants were grown. Of these metals, the data strongly suggest that aluminum (Al) and selenium (Se) are present in washed flour prepared from southern Guam’s cycad seed tissues at elevated levels, especially when the trees are grown in shade. Previous authors have speculated that Al and Se are involved in various ALS outcomes, and our results support this interpretation.

## 1. Introduction

The neurological spectrum disorder of Guam is known as amyotrophic lateral sclerosis-parkinsonism dementia complex (ALS-PDC) and remains one of the most mysterious neurological disorders of the last 100 years. The disorder which was once highly prevalent on Guam combines components of three neurological diseases: ALS, parkinsonism, and dementia which could occur with either of the first two. The ALS aspect was first described by Zimmerman, a United States Navy neurologist [1]. The observations concerning ALS spurred a wave of interest that included Drs. Leonard Kurland and Donald Mulder who went on to describe PDC [2]. Subsequent observations of PDC revealed similarities to progressive supranuclear palsy [3].

The peak expression of ALS-PDC occurred in the 1950s and declined considerably during the 1960s and thereafter [4]. While ALS-PDC could be found throughout the island and on the neighboring island of Rota, various “hotspots” of disease were observed in Guam’s volcanic south. Assessments of the spatial patterns of disease incidence led to widespread belief that localized ephemeral increases in exposure to environmental agent(s) explained causation of the enigmatic disease [5,6].

A number of hypotheses were put forward to account for ALS-PDC. A dominant hypothesis was described as the “cycad” hypothesis which arose from the traditional practice of making flour derived from cycad seeds which have long been known to contain neurotoxins [7]. The hypothesis was founded on a temporary increase during the 1940s of exposure to toxins within the local cycad known as *Cycas micronesica* K.D. Hill due to events associated with World War II, followed by a rapid replacement of traditional foods by imported foods [8,9,10]. The lag between these years and the peak incidence of ALS-PDC comprised the preclinical time that followed the acute environmental insult. The proposed link of ALS-PDC to cycad consumption spurred a hunt for all potential toxins that may have been causal. Several secondary metabolites have been heavily studied in this regard, but to date none of them have been proven to play a causal role on their own [6,11,12,13,14,15]. Exposure to aluminum (Al) found in geological and water substrates has also been proposed as an explanation for the spatiotemporal hotspots of the disease, primarily because Al availability is greatest in southern Guam where the epicenter of ALS-PDC incidence was at an all-time peak [16,17,18]. The metalloid selenium (Se) has not been discussed in the Guam research, but has been linked to ALS in other regions [19].

We conducted extensive field survey work on *C. micronesica* seed production and chemistry, and one of our investigations employed a landscape ecology approach to determine spatial distribution of several cycad sterol compounds in Guam [20]. In an attempt to revisit the Al hypothesis, we set out to determine if cycad seed flour samples from this former investigation contained appreciable amounts of metals and metalloids. We focused on the cycad habitat that was supported by volcanic soils in this preliminary investigation. We first hypothesized that flour derived from *C. micronesica* gametophyte tissues from trees in these acid soils would contain appreciable Al and Se content. Because incident light level is known to influence metal content of *C. micronesica* leaves [21], we also hypothesized that light exposure of the source trees would influence gametophyte metal content. We predicted our results would reveal that the historical dismissal of metal exposure as partly causal of ALS-PDC was premature and that the manner in which the assemblage of neurotoxic secondary metabolites in cycad seeds may combine with toxic metals in those same seeds requires further investigation.

## 2. Materials and Methods

*Cycas micronesica* seeds were harvested from a southern Guam habitat in February 2005 as part of an extensive neurotoxin research project. The theoretical foundation for this project was previously reported [22]. The habitat we selected was a ravine forest supported by acidic soils of volcanic origin, and was described as Site I in Marler et al. [20]. These soils constrain plant health with aluminum toxicity and calcium deficiency [23]. The seeds were a homogeneous 22 months in age. The gametophyte tissue was extracted from seeds, and prepared in a manner that aligned with traditional flour preparation. The only exception was that the tissue was initially frozen at −40 °C and lyophilized prior to proceeding with the flour preparation. All samples were stored at −80 °C until we used these stored samples to determine seed gametophyte metal concentrations in August 2019.

The plant and habitat metadata that were recorded at the time of seed sampling were interrogated to locate samples from trees with the widest range of sunlight exposure. This enabled the selection of 14 samples that were derived from trees within a range in sunlight exposure of 11.1% to 83.2%. The original light data were determined with hemispherical digital photographs using a Nikon fisheye lens capturing the canopy above each tree. The percent open sky was quantified from each image with digital software (Regent Instruments, Inc., Saint-Foy, Quebec, QC, Canada). The tree stems were 232 ± 11 cm in height, and there were 48 ± 3 leaves per tree. 

The pulverized gametophyte samples were digested by a microwave system with nitric acid and peroxide, then metals were quantified by inductively coupled plasma optical emission spectroscopy (Spectro Genesis; SPECTRO Analytical Instruments, Kleve, Germany) [24]. The elements included the essential nutrient nickel; the beneficial elements aluminum, cobalt, and selenium; and the metals arsenic, cadmium, chromium, and lead. This procedure quantified total metal content within the tissue, regardless of whether the metals were free ions or sequestered in secondary metabolites. 

The data were sorted into four light categories as 11−20%, 21−40%, 41−60%, or 61−83%. The data did not conform to prerequisites for use of parametric analytical methods. Therefore, the data were subjected to the Kruskal–Wallis *H* test (SAS Institute, Cary, NC, USA) to determine differences among the four light categories for each of the metals and metalloids. For significant metals and metalloids, we used regression analysis (SAS Institute), to determine the influence of incident light level on gametophyte concentration of each metal/metalloid and the sum of the quantified metals and metalloids. Several metals were not influenced by shade level, but the metals that were significantly influenced by shade were all characterized by linear regression. The percentage discrepancy among the shade levels was determined by a variability index for each of the eight metals or metalloids by calculating with ((maximum − minimum)/maximum) × 100.

## 3. Results

**Figure 1:**
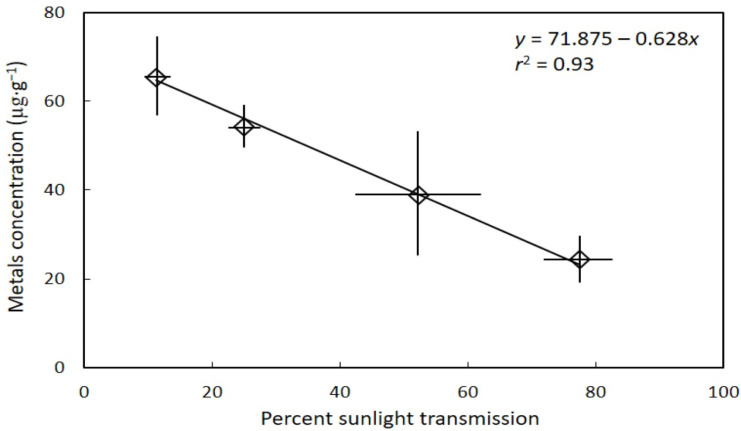
The influence of habitat sunlight transmission on *Cycas micronesica* seed gametophyte metal concentration. Total metals were the sum of aluminum, arsenic, cadmium, chromium, cobalt, lead, nickel, and selenium. Mean ± standard deviation.

The total metal/metalloid concentration of gametophyte tissue was significantly influenced by light (*H* = 8.824, *p* = 0.032), and declined as percent sunlight transmission increased (Figure 1). The predicted metal concentration ranged from about 65 µg·g^−1^ at 11% sunlight transmission to about 20 µg·g^−1^ at 83% sunlight transmission, representing a 3.3-fold difference among the light levels.

**Figure 2:**
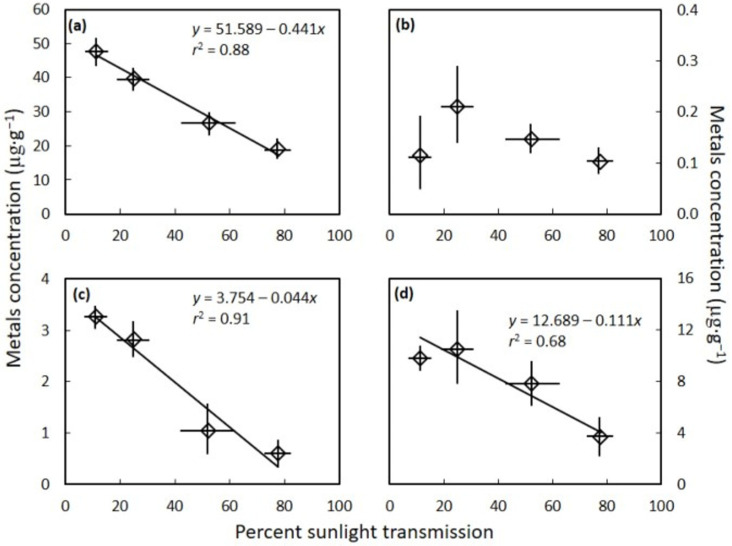
The influence of habitat sunlight transmission on *Cycas micronesica* seed gametophyte metal concentration. (**a**) aluminum; (**b**) cobalt; (**c**) nickel; and (**d**) selenium. Mean ± standard deviation.

The *C. micronesica* seed gametophyte tissue concentrations of the essential metal nickel (*H* = 9.952, *p* = 0.019), and the beneficial elements aluminum (*H* = 11.020, *p* = 0.012) and selenium (*H* = 4.467, *p* = 0.035) differed among the four light categories. These three elements declined in concentration as percent sunlight transmission increased (Figure 2). In contrast, the level of light exposure did not influence the concentration of the beneficial metal cobalt (*H* = 3.390, *p* = 0.335).

**Figure 3:**
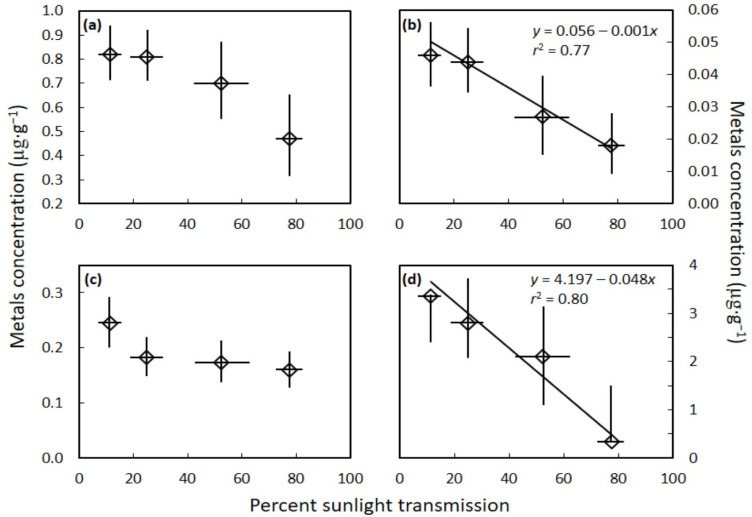
The influence of habitat sunlight transmission on *Cycas micronesica* seed gametophyte metal concentration. (**a**) arsenic; (**b**) cadmium; (**c**) chromium; and (**d**) lead. Mean ± standard deviation.

The *C. micronesica* seed gametophyte tissue concentrations of the metals cadmium (*H* = 7.895, *p* = 0.048) and lead (*H* = 8.333, *p* = 0.040) differed among the light categories, and declined as percent sunlight transmission increased (Figure 3). In contrast, the metals arsenic (*H* = 7.395, *p* = 0.060) and chromium (*H* = 6.333, *p* = 0.096) were not influenced by incident light level. 

The mean concentration of each metal was highly contrasting, with aluminum exhibiting values up to 55 µg·g^−1^ and comprising most of the total metal concentration. Selenium exhibited the second highest concentration, with values up to 15 µg·g^−1^. Lead and nickel were also influential in defining the total metal concentration range, with concentrations up to about 4 µg·g^−1^ in the most shaded trees. The remainder of the metals did not exceed 1 µg·g^−1^ in concentration for any of the sampled trees. These metals and metalloids were present in the cycad seed flour in the order Al > Se > Pb > Ni > As > Cr > Co > Cd.

The variability index varied greatly among the metals (Table 1). Arsenic and chromium concentrations varied the least among the shade levels. Lead and nickel varied the most among the shade levels.

## 4. Discussion

Attempts to understand the spatiotemporal patterns of ALS-PDC in Guam and Rota have pursued the identification of localized exposure to an environmental toxin in southern Guam which occurred antecedent to the 1950s [8,9,10]. The temporary increase in reliance on *C. micronesica* seeds as a source of starch in the human diet during World War II has been the most plausible hypothesis. Our data show that metals and metalloids may be highly concentrated in the gametophyte of *C. micronesica* seeds grown specifically in southern Guam. The elements Al and Se accounted for 90% of the total metal content, results that confirm our first hypothesis. The gametophyte tissue was washed repeatedly in conformity with traditional cycad flour preparation, so the quantified metals were contained in forms that were not water-soluble. The greatest concentrations of metals and metalloids occurred in seeds that were harvested from trees grown in shaded forest microsites. Seeds from trees grown in 11% sunlight transmission contained more than three times the content of metals and metalloids as seeds from trees grown in 83% sunlight transmission, confirming our second hypothesis. 

These data are congruent with those of Yanagihara et al. [17] who reported high Al levels in the southern Guam environment. Of the eight elements we evaluated, Al showed the highest concentrations, results in keeping with Al’s high levels in the Earth’s crust generally and particularly in areas where acid soils predominate. Al is also a known toxin, particularly a neurotoxin, and Al-induced neurodegeneration can be seen in in vivo models of neurological disease, notably Alzheimer’s disease and ALS [25,26,27,28,29]. The second most abundant metal or metalloid in our samples was Se. Exposure to this metalloid may also be correlated with incidence of ALS [19]. It is thus from the considerations of amount and known impact on the nervous system that the current data on Al and Se may be germane for developing a greater understanding of the factors that may have contributed to ALS-PDC. We believe these metals may combine with neurotoxic secondary metabolites to provide new insights into an additive or synergistic effect regarding neurotoxicity of cycad seed flour. 

Our findings come after decades of pursuit of evidence for the cycad hypothesis, which arose from the studies of Dr. Margaret Whiting linking consumption of the seeds of the local cycad (*Cycas micronesica* K.D. Hill) [7]. The link to cycad consumption spurred a hunt for any possible toxins that may have been causal. The focus of these investigations was on various secondary metabolites. Key studies looked at cycasin, an amino sugar which is converted after consumption to the toxin methylazoxymethanol (MAM), β-methyl amino alanine (BMAA), and β-sitosterol β-D glucoside (BSSG) [11,13,15,30]. Of these, MAM appeared as a genotoxin that did not generate key neurological features of the disease within model in vivo systems [8]. BMAA does sometimes produce ALS-PDC-like outcomes, but the cumulative results are less conclusive. BSSG also induced ALS-PDC like outcomes in some studies, albeit of different natures in mice and rats [12,14]. 

The potential role of Al in neurodegeneration in Guam is more complex, partly because the look at its role in ALS-PDC has been sporadic. Al is neurotoxic, but whether it was found on Guam in high concentrations has been in dispute. One report verified high levels of Al in the southern Guam environment [17], but later studies did not confirm the earlier reports [31]. The heterogeneity in results reported among the studies may have been partly responsible for discontinuing the pursuit of the role of Al as a possible source of ALS-PDC. We suggest the lack of focus on habitat and plant characteristics during sampling protocols in the previous field work may explain the differences reported by various research teams, a limitation to the historical ALS-PDC research we have discussed before [22].

Numerous studies have used spatial analysis techniques to look at geographic clusters of ALS in attempts to identify localized environmental exposures. For example, a recent hypothesis was put forward indicating aerosol exposure to cyanobacteria neurotoxins may account for some cases of localized ALS [32]. For Guam’s ALS-PDC case study, the search not only requires identification of a toxin that was localized in southern Guam, but the search also needs to factor in an ephemeral increase in environmental exposure some time antecedent to the peak ALS-PDC incidence in the 1950s [8,9,10]. Our reporting of elevated Al and Se in cycad seeds from trees grown in the shade in southern Guam soils illuminates the possibility of inhalation of cycad-derived Al and Se in the wind-borne pollen within these same forest communities. Indeed, *C. micronesica* pollen cones employ wind to vector some of the pollen [33]. The indigenous peoples of Guam took temporary refuge in the forests during the Japanese occupation of Guam from 1941 to 1944 [10], and increased exposure to air-borne cycad pollen would have been temporarily unavoidable during these conditions. The potential for *C. micronesica* pollen to vector Al and Se has not been determined. 

Our revisitation of the potential link between cycad-derived metal exposure and ALS-PDC comes at a time of increased contamination of human foods by heavy metals. Indeed, environmental pollution of metals and metalloids has emerged as a contemporary concern that impacts food security for much of the world [34,35,36,37,38]. As a result, the biotoxicity of metals in food has attracted attention from policy makers, health organizations, and the research community. Most of the geographic regions where this research has focused are where urbanization, industrialization, or mining enterprises generated the increases in heavy metal contamination of foods [39,40]. Our Guam case study is useful for reminding these stakeholders that exposure to metals in vegetative sources of food may occur in the absence of anthropogenic causes of environmental metal accumulations.

Our report of the influence of light availability on cycad seed metal content adds to a growing body of literature on how light availability influences the ionome and metabolome of edible portions of plants. For example, shade and exposure to Cd influence the metabolic profile of the medicinal *Perilla frutescens* (L.) Britt. [41]. Direct exposure of developing *Styrax tonkinensis* (Pierre) Craib ex Hartwich fruit to sunlight changed secondary metabolites of the reproductive structures when compared to shaded fruit [42]. Nitrate may be the most studied component of the plant ionome-metabolome that is influenced by light availability during plant growth. This ubiquitous source of oxidized nitrogen is rapidly detoxified by plants to less oxidized forms, but only if light availability is sufficient to drive the enzymatic processes. In this case, light availability prior to harvest determines if leafy vegetables meet the standards of food safety [43,44,45,46,47,48]. 

These findings point out future directions of Guam research. First, habitats in more of Guam’s soils should be the subject of cycad gametophyte metal investigations. We focused on a single southern Guam sub-population of *C. micronesica* for this preliminary investigation because the habitat soils were of volcanic origin and exhibited the characteristics that cause Al toxicity of plants. For example, the soil was characterized by low pH and Ca content [23]. Most of Guam’s cycad sub-populations are located on karst soils which are characterized by high pH and Ca content, so more research is needed to determine the risk level for Al and Se exposure from ingesting flour derived from cycad seeds harvested in these other non-volcanic habitats. Second, the genetic structure of the Guam population of *C. micronesica* is characterized by genetically distinct sub-populations exhibiting allopatric distribution [49]. When genotypes from northern Guam were grown in these southern Guam soils, mortality reached 90% in less than 15 years [50]. More sub-population genetics research may reveal if genetic differences among the allopatric sub-populations are partly causal of the elevated Al and Se content in gametophyte tissues in the southern Guam sub-population. The nutritional components of cycad seed flour have been reported for various cycad species [51,52,53,54,55]. To our knowledge, we are the first to report metals and metalloids in appropriately sampled cycad seed flour for the purpose of illuminating anti-nutritional components.

## 5. Conclusions

The potential role of Al exposure in food derived from *C. micronesica* seeds was dismissed in past research programs. We have demonstrated for the first time that Al and Se content can be substantial in *C. micronesica* seed gametophyte tissues in forests nearby the historical ALS-PDC hotspot, and that shaded growth conditions increase content of these and other metals and metalloids. Our findings provide new insights that suggest the historical dismissal of Al as an environmental toxin involved in ALS-PDC was premature and that the neurotoxicity of organic compounds in cycad seeds combined with co-occurring metals requires further investigation.

## Figures and Tables

**Table 1:** The level of plasticity in *Cycas micronesica* seed gametophyte concentration among eight metals. Variability index was calculated with ((maximum − minimum)/maximum) × 100.

Metal	Variability Index
Aluminum	71%
Arsenic	63%
Cadmium	83%
Chromium	60%
Cobalt	93%
Lead	99%
Nickel	96%
Selenium	92%

## Data Availability

Data available upon request.
